# Characterization of Pathogenic *Vibrio parahaemolyticus* Isolated From Fish Aquaculture of the Southwest Coastal Area of Bangladesh

**DOI:** 10.3389/fmicb.2021.635539

**Published:** 2021-03-08

**Authors:** Abu Baker Siddique, M. Moniruzzaman, Sobur Ali, Md. Nayem Dewan, Mohammad Rafiqul Islam, Md. Shafiqul Islam, Mohammed Badrul Amin, Dinesh Mondal, Anowar Khasru Parvez, Zahid Hayat Mahmud

**Affiliations:** ^1^Laboratory of Environmental Health, Laboratory Sciences and Services Division, International Centre for Diarrhoeal Disease Research, Bangladesh, Dhaka, Bangladesh; ^2^Department of Microbiology, Jahangirnagar University, Dhaka, Bangladesh

**Keywords:** *Vibrio parahaemolyticus*, aquaculture, rabbit ileal loop, histopathology, trh

## Abstract

*Vibrio parahaemolyticus* is a major foodborne pathogen responsible for significant economic losses in aquaculture and a threat to human health. Here, we explored the incidence, virulence potential, and diversity of *V. parahaemolyticus* isolates from aquaculture farms in Bangladesh. We examined a total of 216 water, sediment, *Oreochromis niloticus* (tilapia), *Labeo rohita* (rui), and *Penaeus monodon* (shrimp) samples from the aquaculture system where 60.2% (130/216) samples were positive for *V. parahaemolyticus*. Furthermore, we identified 323 *V. parahaemolyticus* strains from contaminated samples, 17 of which were found positive for *trh*, a virulence gene. Four isolates out of the 17 obtained were able to accumulate fluid in the rabbit ileal loop assay. The correlation between the contamination of *V. parahaemolyticus* and environmental factors was determined by Pearson correlation. The temperature and salinity were significantly correlated (positive) with the incidence of *V. parahaemolyticus*. Most of the pathogenic isolates (94.1%) were found resistant to ampicillin and amoxicillin. O8: KUT was the predominant serotype of the potentially pathogenic isolates. ERIC-PCR reveals genetic variation and relatedness among the pathogenic isolates. Therefore, this region-specific study establishes the incidence of potential infection with *V. parahaemolyticus* from the consumption of tilapia, rui, and shrimp raised in farms in Satkhira, Bangladesh, and the basis for developing strategies to reduce the risk for diseases and economic burden.

## Introduction

The Gram-negative, facultative anaerobic bacterium *Vibrio parahaemolyticus* is usually present in tropical and temperate coastal waters, as well as in shrimp aquaculture (Heredia et al., 2009). It is indigenous to the estuarine ecosystem and is associated with human gastrointestinal disease, wound infections, and septicemia (Joseph et al., 1982; Wong et al., 2000). *V. parahaemolyticus* has been considered an etiological agent of foodborne-related illness throughout the world from the very beginning of its discovery (Yeung and Boor, 2004). Consumption of raw, parboiled, or contaminated seafood, including shrimp, prawn, fish, and shellfish, is the most common reason for human gastrointestinal infections (Yeung and Boor, 2004; Mahmud et al., 2007; Su and Liu, 2007; Sala et al., 2009; Khouadja et al., 2013; Khimmakthong and Sukkarun, 2017). A growing number of *V. parahaemolyticus* transmissions and outbreaks engendered by strain members of a pandemic clonal complex have been witnessed all through the world from the early 90s (DePaola et al., 2000; Chowdhury et al., 2004; Ansaruzzaman et al., 2005; González-Escalona et al., 2005; Martinez-Urtaza et al., 2005; Quilici et al., 2005; Fuenzalida et al., 2006).

Vibrio-related infections caused by *V. parahaemolyticus*, which eventually turn into an epidemic, have been observed in Asia, Europe, the United States, Peru, and Chile in the past few decades. These outbreaks were linked with rare, gradual seawater temperature rises along the shoreline (Velazquez-Roman et al., 2014). Several studies performed worldwide implicate environmental factors such as turbidity, temperature, water salinity, amounts of organic matter, and suspended chlorophyll, among others, in the distribution of *V. parahaemolyticus* and the abundance of the species (López-Hernández et al., 2015). Although the mechanism of *V. parahaemolyticus* infection in humans is not well known yet, two hemolysins are broadly accepted as pathogenicity indicators, which are the thermostable direct hemolysin (TDH) and the tdh-related hemolysin (TRH) (West et al., 2013; Jones et al., 2014; López-Hernández et al., 2015). tdh is a protein that helps in pore formation, which has been implicated in the invasion of bacteria, and *trh* plays a major role in virulence. Many *V. parahaemolyticus* clinical isolates have *trh* and/or *tdh*; however, a comparatively limited quantity of environmental isolates harbor such genes (López-Hernández et al., 2015; Leoni et al., 2016).

There are 71 K antigens and 13 O antigens that have been recognized and considered related to gastroenteritis by the Serological Typing Committee of *V. parahaemolyticus* (Ishibashi et al., 2000). Typically, in the outbreaks, a wide number of serovars (O3:K6, O4:K68, O1:K25, O1:KUT, O1:K26 etc.) are present (Velazquez-Roman et al., 2014). Post-1996, the growing occurrence of gastroenteritis was associated with the pandemic serotype O3:K6 in most of the world (Okuda et al., 1997; Bag et al., 1999). In recent years, the correlation of this serotype O3:K6 with most of the *V*. *parahaemolyticus* infections in the United States, South Korea, Thailand, Japan, Laos, Taiwan, and India indicates that this species might have had an uncommon food-borne trait and possess the pandemic ability (Bag et al., 1999). Several molecular typing techniques such as random amplified polymorphic DNA (RAPD), ribotyping, pulsed-field gel electrophoresis (PFGE), and multi-locus sequence typing (MLST) (Bag et al., 1999; Marshall et al., 1999; Wong et al., 1999; González-Escalona et al., 2008; Yang et al., 2008) have been valuable for illustrating the genetic diversity at the strain level (Olive and Bean, 1999). For the subtyping of the *V. parahaemolyticus* species containing strongly conserved repetitive intergenic consensus sequences, ERIC-PCR has been shown to be efficient (De Bruijn, 1992; Chen et al., 2012).

Shrimp export is positioned as one of the leading earning sectors in Bangladesh. Over 40,000 tons of *P. monodon* and *M. rosenbergii* shrimps were exported in 2016 and 2017 along with smaller volume of other species. More than 80% of the shrimp production was shipped to the European Union market, mainly Germany, Netherlands, and Belgium, in 2017. According to the estimation of the fish industry, 5.02 million metric tons of fish production will be achieved within 2020–21. However, the number of detention and rejection cases due to the detection of *V. parahaemolyticus* from Asia is increasing (Sujeewa et al., 2009).

In multiple ecological studies, the relationship of temperature and salinity with *V. parahaemolyticus* has been reported, but the extension of this relationship depends on the region and season (Takemura et al., 2014). Similarly, nutrients, pH, and turbidity were incoherent and depended on the region and the variability of these factors. Therefore, it is necessary to conduct region- and area-specific studies to provide a detailed description of the effect of environmental parameters on *V. parahaemolyticus* concentration (Froelich and Noble, 2016).

Consumption of undercooked and raw seafood is the primary way of *V. parahaemolyticus* exposure and infection. Fishes and shrimps can accumulate the bacterium from the aquaculture prior to their harvesting process. As the incidence of *Vibrio* spp. in the environment is unavoidable, their increasing presence in coastal water and sediment create public health issues for the aquaculture industry and the consumers. Therefore, substantial interest has been grown in understanding the biotic and abiotic factors of *V. parahaemolyticus.* Such data has value for estimating the properties and abundance of the species in aquaculture.

Standing on the abovementioned basic contextual information, we conducted a yearlong field-based study to correlate the abundance of non-pathogenic and pathogenic *V. parahaemolyticus* with seasons and other factors such as temperature, pH, and salinity. In this study, pathogenic strains were further characterized to determine their virulence potential in a rabbit model.

## Materials and Methods

### Sample Types, Sources, and Study Sites

**FIGURE 1:**
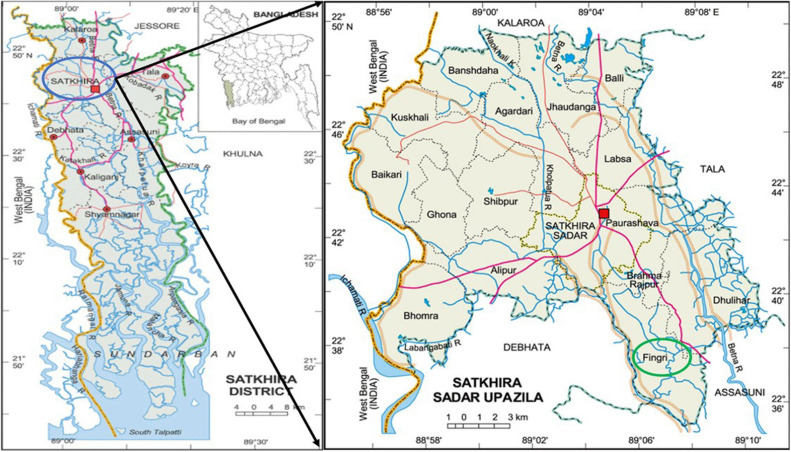
Location of the sample collection sites of the coastal area of Satkhira, Bangladesh. Image courtesy: Banglapedia.

Water, sediment, and three different types of fishes (*Penaeus monodon*—shrimp, *Oreochromis niloticus*—tilapia, and *Labeo rohita*—rui) were collected from three sampling sites located in Satkhira Sadar Upazila of Satkhira district (Figure 1), one of the major shrimp-harvesting areas of Bangladesh. The sampling was conducted from the period of May 2017–April 2018, once a month. The sampling sites were (1) low-saline aquaculture: shallow water (22° 38’ 44.9” N 89° 04’ 47.9” E) (2) brackish aquaculture: enclosed river water (22°39’28.9”N 89°04’30.6”E), and (3) brackish water: flowing river water (22°39’45.9”N 89°04’33.4”E). Water and sediment were taken from all three sites, whereas Tilapia was collected from sites (1) and (2), and rui fish and shrimp were collected only from site (1) and site (2), respectively.

### Sample Collection

All the samples were collected in sterile bottles (Nalgene, United States) of 500 ml and 250 ml and a sterile easy-to-close zipper bag (Fisherbrand^TM^ Whirl-Pak^TM^, United States). Water and sediment samples were taken from three different spots of each site, and three pieces of each category of fishes were picked up in every sampling time to cover the maximum areas of the site according to the American Public Health Association (APHA) (Federation and American Public Health Association, 2005). After collection, samples were shipped to the Laboratory of Environmental Health (LEH) at the International Centre for Diarrhoeal Disease Research, Bangladesh (icddr,b), in an insulated sample-carrying box maintaining the temperature ranging from 4 to 10°C. Then, the samples were processed within 12 h of collection according to the APHA guideline.

### Measurement of Physicochemical Parameters of Water Samples

Salinity and temperature were measured using a conductivity meter (Sens-Ion 5, HACH, United States), and pH of the water samples was measured with a pH meter (Sens-Ion^+^ pH1, HACH, United States) in each site following the manufacturer’s instructions.

### Sample Processing

Inoculation of water and sediment samples on Thiosulfate Citrate Bile Salt Sucrose (TCBS) (DIFCO, BD, United States) agar was done by the spread plate method. Fish samples were washed with sterile 3% NaCl solution to remove surface microorganisms, debris, sand, and slime. Then, the flesh, gut, and gill of the tilapia and rui samples and the flesh and chitin of the shrimp samples were separated aseptically. After that, 5 g of each separated part was homogenized in 45 ml of 3% NaCl solution separately in autoclaved 4 Oz bottles. Then, fish homogenates, water, and sediment samples were enriched in alkaline peptone water (APW) for 18 h. The enrichment broths were then subcultured onto TCBS agar plates and incubated at 37°C overnight. Later on, 4–6 presumptive green or blue-green colonies of 2–3 mm in diameter from each sample were transferred to fresh TCBS agar plates and Chromogenic Vibrio Agar (CVA) (CHROMagar, Paris, France) plates (Hara-Kudo et al., 2001). Isolates showed green to blue green colonies in TCBS, and violet colonies in CVA plates were selected as *V. parahaemolyticus*. Subsequently, the selected strains were inoculated onto Gelatin Agar (GA) plates to see the gelatinase activity for further confirmation. The isolates were preserved at −80°C in LB broth with 30% glycerol in the icddr,b central bio-repository system and in T1N1 soft agar media at room temperature (Alam et al., 2009). For each experiment, the isolates were subcultured on GA plates from the stocks and kept at 37°C for overnight incubation.

### Polymerase Chain Reaction (PCR) Assay

Extraction of chromosomal DNA was done using the boil DNA extraction method according to the procedure reported by Mahmud et al. (2020). Presumptively identified isolates were further confirmed by simplex PCR for the *tlh* gene, a species-specific gene for *V. parahaemolyticus* (Taniguchi et al., 1986; Bej et al., 1999; McCarthy et al., 1999; Jones et al., 2012), and the presence of 12 virulent genes was also examined. The presence of all these genes was tested by performing simplex PCR. PCR assays were carried out to detect *tdh* and *trh* genes described by Bej et al. (1999); the *GS*, *PGS*, *orf-8*, and *HU-*α genes described by Li et al. (2016); and the *ure*, *Mtase*, *VPA 1339* (*escC*, *vscC2*), *VPA 1346* (*yopP*, *vopP*, *vopA/P*), *VPA1321* (*vopC*), and *VPA 1376* genes reported in Caburlotto et al. (2009). All the primers’ sequence and band size are listed in Table 1. The PCR-amplified products were separated in an agarose gel of 1.5% stained in MIDORI green advance and visualized under UV light with a GelDoc Go imaging system (Bio-Rad, United States).

**TABLE 1:** Primer list used in this study.

Primer name	Primer sequence (5′–3′)	Amplicon size (bp)	References
***tlh F***	AAAGCGGATTATGCAGAAGCACTG	450	Bej et al., 1999
***tlh R***	GCTACTTTCTAGCATTTTCTCTGC		
***tdh F***	GTAAAGGTCTCTGACTTTTGGAC	269	Bej et al., 1999
***tdh R***	TGGAATAGAACCTTCATCTTCACC		
***trh F***	TTGGCTTCGATATTTTCAGTATCT	500	Bej et al., 1999
***trh R***	CATAACAAACATATGCCCATTTCCG		
***orf8-F***	GTTCGCATACAGTTGAGG	700	Nasu et al., 2000
***orf8-R***	AAGTACACAGGAGTGAG		
***GS-PCR- F***	FTAATGAGGTAGAAACA	651	Matsumoto et al., 2000
***GS-PCR- R***	ACGTAACGGGCCTACA		
***PGS-PCR-F***	TTCGTTTCGCGCCACAACT	235	Okura et al., 2004
***PGS-PCR-R***	TGCGGTGATTATTCGCGTCT		
***HU-*α *-F***	CGATAACCTATGAGAAGGGAAACC	474	Williams et al., 2004
***HU-*α *-R***	CTAGAAGGAAGAATTGATTGTCAAATAATG		
***ure F***	CTTGTCATCGGGTGTCACTA	464	Caburlotto et al., 2009
***ure R***	GATGTTAGGTTCACCTACTGACT		
***vscC2 F***	GCGGTCTATTGCTATCCT	362	Caburlotto et al., 2009
***vscC2 R***	TCTTGGTATTGATAGTGGGTG		
***vopP F***	CGTCCAACTCTATTGTTGTG	393	Caburlotto et al., 2009
***vopP R***	CAATGTTGGCTATTCGGTTG		
***MTase F***	GTCTTGTCGAATAGAACTCTGA	683	Wang et al., 2006
***MTase R***	TAAGCTCCAAAATCCATACG		
***vopC F***	CAGAGTTGGTTTCGCAG	579	Caburlotto et al., 2009
***vopC R***	CTGGTACGCCTCTTGGACAG		
***VPA1376 F***	GCTCTCCTTGGTACCAATCAC	1067	Caburlotto et al., 2009
***VPA1376 R***	CTGGGATCTTGATGTCAAGGT		

### Species Confirmation by VITEK2

All 17 *tlh* and *trh* gene-positive *V. parahaemolyticus* isolates were further confirmed by the VITEK 2 system (bioMérieux, Marcy I’Etoile, France) using a VITEK 2 GN ID card. *V. parahaemolyticus* (ATCC BAA-238) was used as a positive control for the identification in this system. For VITEK 2 assays, pure isolates were streaked on GA plates and incubated at 37°C overnight. 1–3 isolated colonies were selected from each GA plate and suspended in saline for preparation of inoculum to obtain an absorbance of ∼0.5 McFarland Units before being subjected to VITEK 2 analysis.

### Antimicrobial Susceptibility Testing (AST) and Calculation of Multiple Antibiotic Resistance (MAR) Indexes

By following the recommendation of the Clinical and Laboratory Standards Institute (CLSI) guidelines (Patel et al., 2015), the pattern of antibiotic susceptibility for 17 *trh*-positive isolates was obtained by disk diffusion technique. A bacterial suspension with turbidity comparable to 0.5 McFarland standard was made from bacterial inocula taken from overnight cultured Gelatin Agar (GA) plate and swabbed onto the Mueller-Hinton Agar (MHA) (Difco Detroit, MI, United States). At 37°C, the plates were then kept for overnight incubation aerobically. Total 15 antibiotic discs (Oxoid, United States) including amoxicillin (AML; 10 μg), ampicillin (AMP; 10 μg), cefotaxime (CTX; 30 μg), ceftriaxone (CRO; 30 μg), cefixime (CFM; 5 μg), cefoxitin (FOX; 30 μg), ciprofloxacin (CIP; 5 μg), gentamicin (CN; 10 μg), nalidixic acid (NA; 30 μg), streptomycin (S; 10 μg), tetracycline (TE; 30 μg), trimethoprim/sulfamethoxazole (SXT; 25 μg), piperacillin-tazobactam (TZP; 110 μg), imipenem (IPM; 10 μg), and meropenem (MEM; 10 μg) were used. The inhibition zone surrounding each disk was determined in millimeter (mm) and documented. As per CLSI guidelines, each bacterial species was categorized as resistant (R), intermediate (I), or susceptible (S) (Clinical and Laboratory Standards Institute [CLSI], 2017). The *Escherichia coli* ATCC 25922 strain was used as a negative control. Calculation of the MAR index was made using formula x/y, where x is the antimicrobial number to which an isolate became resistant and y indicates the antimicrobial number to which the isolate got exposed (Krumperman, 1983).

### Serotyping

Serotyping of *V. parahaemolyticus* toxigenic strains was performed utilizing commercially available *V. parahaemolyticus* antisera test kit (Denka Seiken, Tokyo, Japan), as defined by Shinoda et al. (1983). The strains were first grown on 3% NaCl-containing Luria-Bertani agar. After incubating for 24 h at 37°C, one loopful inoculum was taken and mixed with 1 ml of normal saline. After boiling for 2 h according to the manufacturers’ instruction, the cell suspension aliquots containing normal saline were utilized for serotyping on the basis of O antigen. The leftover cell suspension (not boiled) was used for K antigen-dependent serotyping.

### ERIC-PCR and Genetic Fingerprinting Analysis

To determine the clonal relationship among the isolates based on Enterobacterial Repetitive Intergenic Consensus (ERIC) sequences PCR, two primer sequences ERIC1 (5’-3’ATGTAAGCTCCTGGGGATTCAC) and ERIC2 (5′–3′ AAGTAAGTGACTGG GGTGAGCG) (Versalovic et al., 1991) were used. The conducted amplification conditions were reported by Rivera et al. (1995). Separation of 20 μl PCR product on a 2% agarose gel was performed at 54 volts for 120 min. 1 kb Plus ladders from Invitrogen (Thermo Fisher Scientific, United States) were used as DNA marker. The gel imaging analysis was performed by BioNumerics software (version 4.5) (Applied Maths, Belgium) using the dice coefficient and unweighted-pair group method where average linkages were used to create dendrogram with 1.0% tolerance values.

### Rabbit Ileal Loop Study

The rabbit ileal loop assay was performed to examine the induction of fluid accumulation as previously described (Twedt et al., 1980), with minor modifications. The experiment was carried out in duplicate with 1.8–2.2 kg adult New Zealand white rabbits. The pathogenic strains of *V. parahaemolyticus* (10^8^ CFU/ml) were injected into the ligated ileal loops of rabbits, which was followed by measurement of the fluid accumulation in each loop at 18 h after injection. The fluid accumulation (FA) ratio was calculated by dividing fluid accumulation (in ml) in each loop by the length (in cm) of the loop. Phosphate-buffered saline (PBS) was used as a negative control, and *V. cholerae* O1 was used as a positive control in this study. Animal Experimental Ethics Committee of International Centre for Diarrhoeal Disease Research, Bangladesh has approved this rabbit ileal loop study (permit number PR-20114).

### Histopathology

Histopathological examination of the intestinal tissue of the rabbits was also performed to observe the histopathological features of the intestinal wall. Each ligated intestinal loop was washed in phosphate-buffered saline (PBS), and appropriately 2 cm in length was sectioned and fixed in 10% formalin for histopathological examination under a light microscope. Tissues were dehydrated in a series of graded alcohols, further processed, embedded in paraffin, and mounted into paraffin blocks. Staining of the 4 μm tissue sections was performed with hematoxylin and eosin (H&E). The protocols for H&E staining were as described by Bancroft and Gamble (2008).

### Statistical Analysis

Pearson correlation analysis was done to observe the relationship between *V. parahaemolyticus* contamination and physicochemical parameters. On the other hand, logistic regression analysis was performed to see the relationship between *trh*-positive *V. parahaemolyticus* and physicochemical parameters. The odds ratio was calculated from the coefficients of the model to identify significant risk factors. The desired statistical significance level was set at 0.05. The analysis was performed by programming language R (R Core Team, 2013) and statistical software package STATA 13 (StataCorp, 2014).

## Results

### Variations Among the Sites and Samples

A total of 216 samples from 3 different sites [96 samples from site (1), 96 samples from site (2), and 24 samples from site (3)] were analyzed where 60.2% (130/216) samples were positive for *V. parahaemolyticus*, and 7.69% (10/130) of total contaminated samples contained *trh* gene. Later on, from 130 contaminated samples, 323 representative *V. parahaemolyticus* strains were obtained, of which 17 strains were found positive for the trh gene. The prevalence of *V. parahaemolyticus* in contaminated samples was highest in samples collected from site (2) at 81.25% (78/96), closely followed by site (3) at 79.16% (19/24). Site (1) presented the lowest incidence of *V. parahaemolyticus* at 34.38% (33/96). The prevalence of virulence factor, trh, was most prominent in site (3) defined at 12.5% (3/24) followed by site (1) with a 4.17% (4/96) incidence and site (2) at 3.13% (3/96). Among the samples, *V. parahaemolyticus* was most prevalent in sediment 72.2% (26/36) followed by shrimp 69.4% (25/36), tilapia 66.7% (48/72), water 61.1% (22/36), and rui 25% (9/36) (Table 2).

**TABLE 2:** *V. parahaemolyticus* contamination among sampling sites and samples.

Sites	Sampling site 1			Sampling site 2			Sampling site 3			Overall		
Sample type	No. of sample	Contaminated sample	% of contamination	No. of sample	Contaminated sample	% of contamination	No. of sample	Contaminated sample	% of contamination	No. of sample	Contaminated sample	% of contamination
Sediment	12	3	25	12	12	100	12	11	91.7	36	26	72.22
Water	12	3	25	12	11	91.7	12	8	66.7	36	22	61.11
Tilapia	36	18	50	36	30	83.3	−	−	−	72	48	66.67
Rui	36	9	25	−	−	−	−	−	−	36	9	25.00
Shrimp	−	−	−	36	25	69.4	−	−		36	25	69.44
Total	96	33	34.38	96	78	81.25	24	19	79.2	216	130	60.19

**FIGURE 2:**
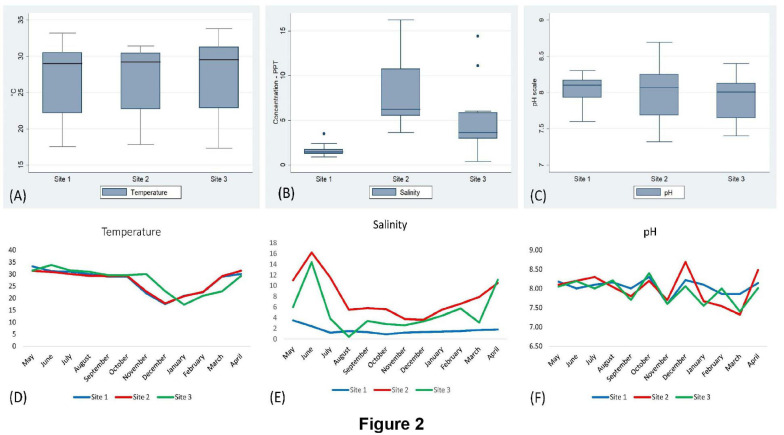
Analysis of physicochemical parameters (temperature, salinity, and pH). Box and whisker plot analysis of temperature **(A)**, salinity **(B)**, and pH **(C)** in sites (1), (2), and (3). Seasonal variation of temperature **(D)**, salinity **(E)**, and pH **(F)** in different sites.

**FIGURE 3:**
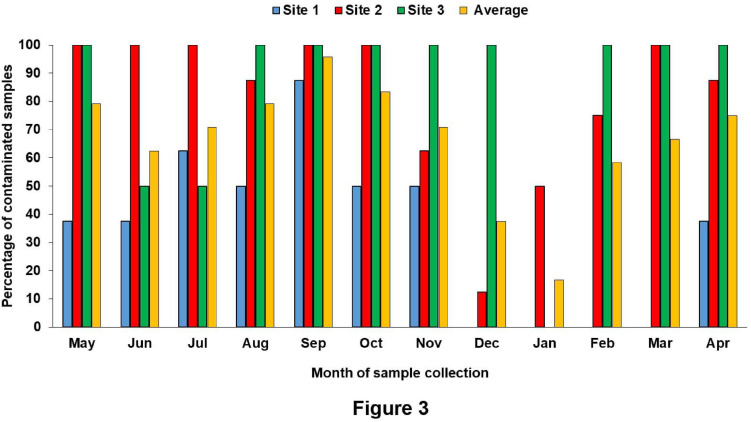
Seasonal distribution of *V. parahaemolyticus* in different sites.

The surface water temperature at three sampling sites recorded from May 2017 to April 2018 was between 17.3 and 33.8°C. The median temperatures at site (1), site (2), and site (3) were 29, 29.25, and 29.5°C, respectively. The salinity and pH of surface water varied from 0.4 to 16.2 ppt and 7.32 to 8.69, respectively (Figure 2 and Supplementary Table S1). During the summer (March–June) and the rainy season (July–October), *V. parahaemolyticus* contamination in the samples was higher as compared to the winter seasons (November–February) in all the three sampling sites (Figure 3). In the case of Pearson correlation analysis, a correlation coefficient of 0.41 for salinity indicates a significant (*p*-value < 0.05) positive relation with *V. parahaemolyticus* contamination, and similarly, the correlation coefficient of 0.45 for temperature gives a significant (*p*-value < 0.05) positive relation with *V. parahaemolyticus* contamination (Table 3). On the other hand, in the case of logistic regression, we observed a significant negative relation between salinity and *trh* positive *V. parahaemolyticus* (odds ratio 0.83, *p*-value < 0.05) (Table 4).

**TABLE 3:** Pearson correlation analysis between *V. parahaemolyticus* and physicochemical parameters.

	Physicochemical parameter	Correlation coefficient	*P*-value
*V. parahaemolyticus* contamination	Salinity	0.41	< 0.05
	Temperature	0.45	< 0.05
	pH	−0.08	> 0.05

**TABLE 4:** Logistic regression analysis of *trh*-positive *V. parahaemolyticus* and physicochemical parameters.

Independent variable	Dependent variable (*V. parahaemolyticus*, trh positive = 1*)*
	Odds ratio (confidence interval)
Salinity	0.83* (0.72, 0.97)
Temperature	0.94 (0.83, 1.06)
pH	2.41 (0.44, 13.32)

*p-value < 0.05, significant at 5% level of significance

### Molecular and Phenotypic Confirmation of the Isolates

**FIGURE 4:**
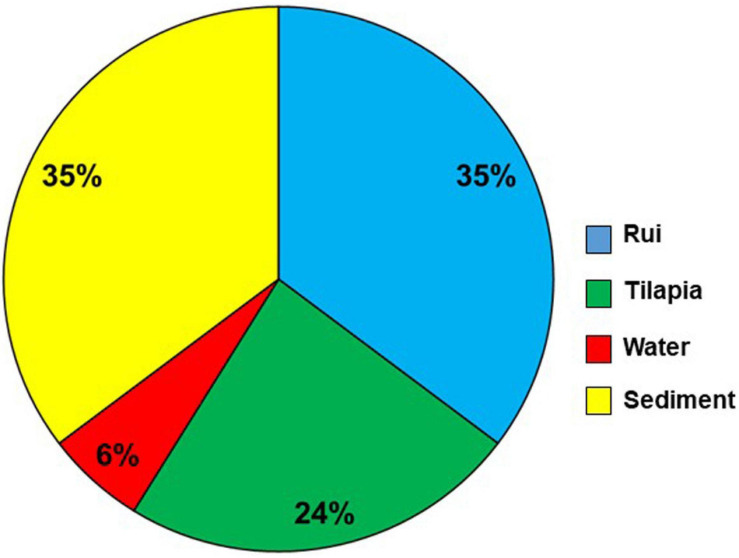
*trh*-positive *V. parahaemolyticus* distribution in different sample types.

The presence of *tlh* (species-specific gene) confirmed the isolates of *V. parahaemolyticus*. The occurrence of 12 virulence and virulence-associated genes was detected by PCR analysis of the isolates. Most of the isolates were negative for the genes tested. However, 5.26% (17/323) isolates were positive for the *trh* gene, a major virulence gene for *V. parahaemolyticus*, considered as pathogenic. All the 17 *tlh* and *trh* gene-positive isolates were further confirmed as *V. parahaemolyticus* species by using the *tlh* species-specific primer (Supplementary Figure S1) and the VITEK 2 system. Among the *trh*-positive isolates, 6 (35%), 6 (35%), 4 (24%), and 1 (6%) were from sediment, rui, tilapia, and water samples, respectively (Figure 4). No *trh*-positive isolate was found from the shrimp sample.

### Antibiogram

Antibiotic susceptibility test (AST) was performed on isolated pathogenic *V. parahaemolyticus* using 15 antibiotics selected from different groups (Supplementary Table S2). Referring to Table 5, *V. parahaemolyticus* isolates were the most resistant toward ampicillin and amoxicillin (94.1%), followed by cefotaxime (29.4%), and ceftriaxone (17.6%). Some of the antibiotics, such as gentamicin and streptomycin, had a slight resistance of about 11.8% and 5.9%, respectively. All other examined isolates were sensitive to imipenem, sulfamethoxazole-trimethoprim, nalidixic acid, tetracycline, and pipercillin-tezobactam. The next-highest susceptibility rates were found for streptomycin (94.1%), meropenem (88.2%), ciprofloxacin (88.2%), gentamicin (82.4%), cefoxitin (82.4%), ceftriaxone (70.6%), and cefixime (64.7%).

**TABLE 5:** Antimicrobial resistance profiles of *V*. *parahaemolyticus* isolates.

Antimicrobial agents	Number (%) of S	Number (%) of I	Number (%) of R
Amoxicillin (AML)	0(0.0)	1(5.9)	16(94.1)
Ampicillin (AMP)	0(0.0)	1(5.9)	16(94.1)
Ceftriaxone (CRO)	12(70.6)	2(11.8)	3(17.6)
Cefotaxime (CTX)	11(64.7)	1(5.9)	5(29.4)
Cefixime (CFM)	11(64.7)	6(35.3)	0(0.0)
Cefoxitin (FOX)	14(82.4)	3(17.6)	0(0.0)
Meropenem (MEM)	15(88.2)	2(11.8)	0(0.0)
Imipenem (IPM)	17(100.0)	0(0.0)	0(0.0)
Nalidixic acid (NA)	17(100.0)	0(0.0)	0(0.0)
Ciprofloxacin (CIP)	15(88.2)	2(11.8)	0(0.0)
Gentamicin (CN)	14(82.4)	1(5.9)	2(11.8)
Streptomycin (S)	16(94.1)	0(0.0)	1(5.9)
Sulfamethoxazole-trimethoprim (SXT)	17(100.0)	0(0.0)	0(0.0)
Tetracycline (TE)	17(100.0)	0(0.0)	0(0.0)
Piperacillin-tazobactam (TZP)	17(100.0)	0(0.0)	0(0.0)

The MAR index was obtained by considering the relation between the number of antibiotics that an isolate is resistant and the total number of antibiotics used. The MAR index range was from 0.07 to 0.27 (Table 6). The maximum MAR index was obtained from two isolates (R06-106, R06-107) from the gut of rui fish, which showed resistance to four antibiotics.

**TABLE 6:** Antibiograms and multiple antimicrobial resistance (MAR) indices of *V. parahaemolyticus* strains.

Antibiotic-resistant pattern	Isolates	Total antibiotic resistance	No. of isolates	MAR index
AML-AMP-CRO-CTX	R06-107	4	1	0.27
AML-AMP-CN-CTX	R06-106	4	1	0.27
AML-AMP-CTX	R06-75, R06-99, R09-14	3	3	0.20
AML-AMP-CN	R06-70	3	1	0.20
AML-AMP-CRO	R06-100, R06-101	3	2	0.20
AML-AMP-S	R11-40	3	1	0.20
AML-AMP	R01-15, R01-54, R01-55, R09-20, R10-16, R12-76	2	6	0.13
AML	R06-88	1	1	0.07
AMP	R06-102	1	1	0.07

### Serotyping Analysis

All the 17 pathogenic *V. parahaemolyticus* isolates were serotyped and categorized into 5 different O groups (O1, O3, O5, O8, and O11). Groups of O8 (47.1%), O5 (23.53%), O11 (11.76%), O3 (11.76%), and O1 (5.88%) were found in this study. Among the isolates, only one was determined by specific K-typing, whereas the rest of the isolates were K-typing untypable. O8: KUT (with eight isolates) was the most common serotype among them. Other O: K serogroups included four O5: KUT, two O11: KUT, one O3: KUT, O1: KUT, and O3: K20 each (Table 7).

**TABLE 7:** List of *trh*-positive *V. parahaemolyticus* isolates.

Serial no.	Strain ID	Source	Site no.	Time of collection	Serology	Resistant profile
1	R01-15	Sediment	3	May 2017	O11: KUT	AML-AMP
2	R01-54	Sediment	2	May 2017	O5: KUT	AML-AMP
3	R01-55	Sediment	2	May 2017	O11: KUT	AML-AMP
4	R06-70	Tilapia (flesh)	1	October 2017	O8: KUT	AML-AMP-CN
5	R06-75	Tilapia (gill)	1	October 2017	O8: KUT	AML-AMP-CTX
6	R06-88	Tilapia (gut)	1	October 2017	O3: KUT	AML
7	R06-99	Rui (gill)	1	October 2017	O8: KUT	AML-AMP-CTX
8	R06-100	Rui (gill)	1	October 2017	O8: KUT	AML-AMP-CRO
9	R06-101	Rui (gill)	1	October 2017	O8: KUT	AML-AMP-CRO
10	R06-102	Rui (gut)	1	October 2017	O8: KUT	AMP
11	R06-106	Rui (gut)	1	October 2017	O8: KUT	AML-AMP-CN-CTX
12	R06-107	Rui (gut)	1	October 2017	O8: KUT	AML-AMP-CRO-CTX
13	R09-14	Sediment	2	January 2018	O1: KUT	AML-AMP-CTX
14	R09-20	Sediment	2	January 2018	O3: K20	AML-AMP
15	R10-16	Water	3	February 2018	O5: KUT	AML-AMP
16	R11-40	Sediment	3	March 2018	O5: KUT	AML-AMP-S
17	R12-76	Tilapia (gill)	2	April 2018	O5: KUT	AML-AMP

### ERIC-PCR

**FIGURE 5:**
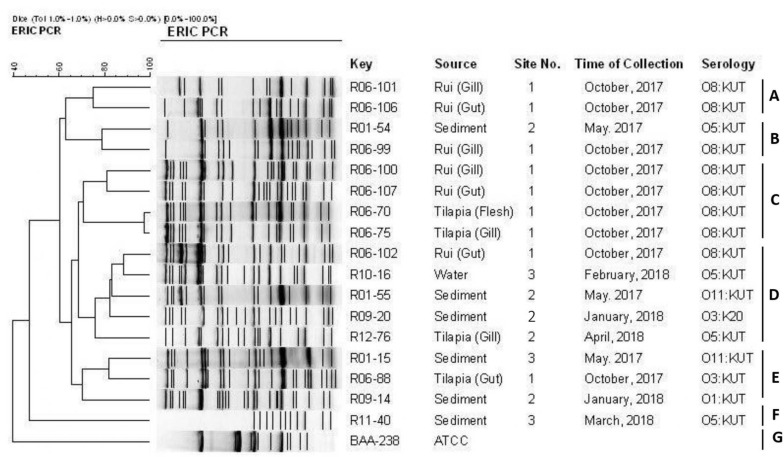
ERIC-PCR DNA fingerprint analysis of *trh*-positive *V. parahaemolyticus* isolates in different samples (fish, sediment, water) from Satkhira, Bangladesh.

ERIC-PCR of the 17 pathogenic isolates resulted in 11–25 amplification bands with a molecular size ranging from 100 to 3,000 bp, where 420, 520, and 1,500 bp were common to most of the isolates. However, considering the similarity coefficient of 0.68, the isolates were divided into 7 clusters designated as A, B, C, D, E, F, and G. Most of the isolates were distributed between the C and D clusters. ERIC-PCR banding patterns showed that all the isolates were genetically diverse except one isolate pair (R06-70 and R06-75). Only one strain (R11-40) from sediment is clustered in group F. The reference strain ATCC BAA-238 was in cluster G alone (Figure 5).

### Ileal Loop Study and Histopathology

**FIGURE 6:**
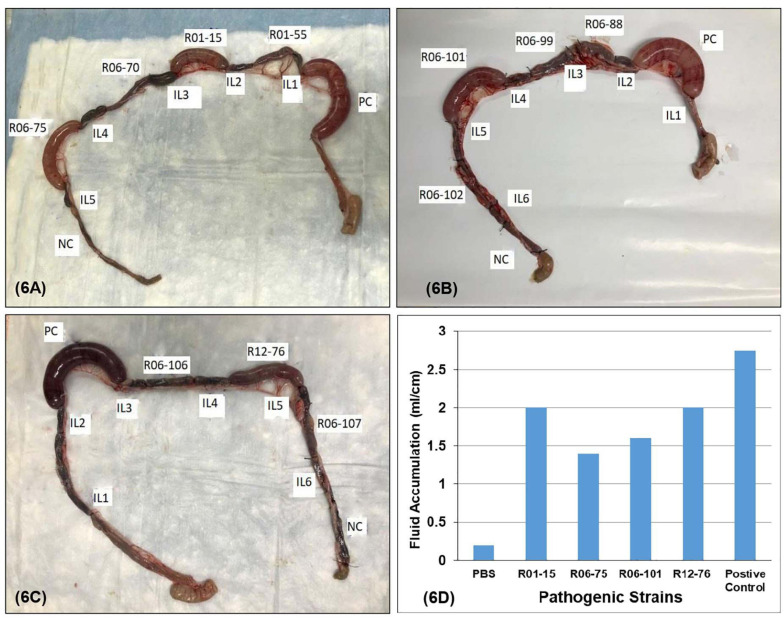
Rabbit ileal loop assay to assess enterotoxigenic activity. Pictorial view of rabbit ileal loop of different environmental strains. Analysis of fluid accumulation of different *V. parahaemolyticus* strain isolated from the environment **(D)**. Rabbit ileal loops were inoculated with 10^8^ CFU/ml of each *trh*-positive *V. parahaemolyticus* strain **(A–C)**. Results are expressed as fluid accumulation (FA; in milliliters) per loop length (in centimeters). PBS and the *V. cholerae* O1 were taken as controls in this assay.

**FIGURE 7:**
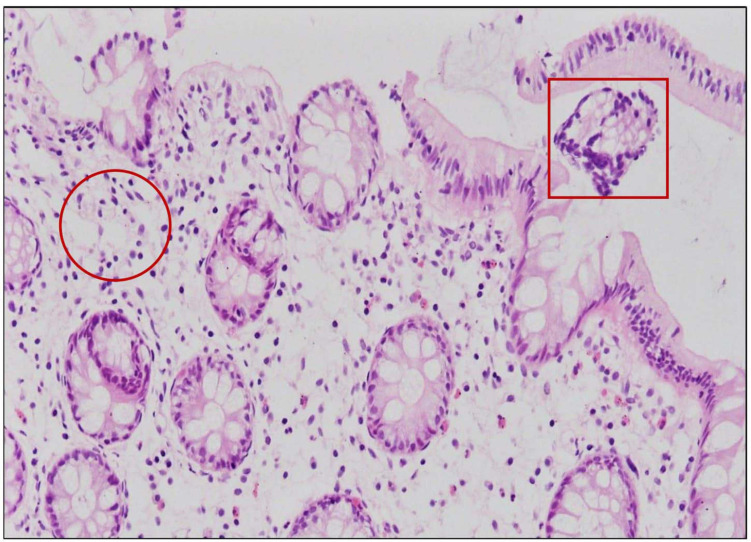
Ileal tissue sections obtained from rabbits infected with 10^8^ CFU/ml of *trh* positive *V. parahaemolyticus* showed severe histopathological changes in the mucosa represented by infiltration with PMN in the lamina propria (inside circle) and degeneration, damage, and sloughing (inside square).

Four out of 17 pathogenic *V. parahaemolyticus* showed fluid accumulation in the rabbit ileal loop. The fluid accumulation (FA) ratio was 2.0, 1.4, 1.6, and 2.2 ml/cm in strains R01-15, R06-75, R06-101, and R12-76, respectively (Figure 6). Among the four strains, R06-101 showed more serosal hemorrhage than the other three strains, characterized by gross bloody mucous in the accumulated fluid. Histopathological features of all four strains showed nearly similar findings, including a moderately degenerated, damaged, and sloughed ileal mucosa (Figure 7; Inside Square); significant/obvious obstruction of blood vessels and infiltration of polymorphonuclear neutrophils (PMN) in the lamina propria and at the crypt epithelium surface were observed at higher magnification (Figure 7; Inside Circle).

## Discussion

The occurrence of *V. parahaemolyticus* has been reported previously in temperate and tropical coastal waters as well as in aquaculture (Heredia et al., 2009; Pui et al., 2014). The incidence of *V. parahaemolyticus* in shrimp, water, fish, and sediments was investigated all year round using cultural and molecular methods. In our study, *V. parahaemolyticus* was present year-round in site (2) and site (3), whereas it was absent in site (1) during the winter-period. Surprisingly, the presence of the pathogenic (*trh*) gene was more frequent during October from the rui fish sample than others. The seasonal and geographical distribution of *V. parahaemolyticus* has been considered to be significantly dependent on temperature in temperate zone shellfish-growing areas (Kaneko and Colwell, 1975; Kiiyukia et al., 1989; Mahmud et al., 2006).

Based on the year-long assessment of water sample salinity gradients, for which sample collection was done from the three study locations, a salinity-dependent area demarcation can then be made. During summer, maximum salinity was encountered in site (2) and site (3) due to an increased brackish water inflow, whereas in site (1), low salinity was present throughout the year as it was collected from underground shallow water, which is not affected by seawater. Contrariwise, minimum salinities in site (2) and site (3) were reported in the rainy season due to heavy rainfalls associated with the blending of floodwater from the nearby regions. At all three study locations, the temperature of water varied consistently, the highest temperature was reported in the summer, followed by the rainy season, and the lowest in the winter; the pH was somewhat unchanged (7.99 ± 0.31) within its intrinsic alkaline status. However, in the rainy season, the maximum alkalinity may be due to the floodwater inflow from the adjacent areas containing a significant amount of organic residues.

Statistically, the salt tolerance potential (at a salt concentration of 2–10%) can be due to a crucially significant positive correlation between salinity and *V. parahaemolyticus* (Table 3; Caburlotto et al., 2010; Johnson et al., 2010; Whitaker et al., 2010) but a significant negative association was found between salinity and the pathogenic *V. parahaemolyticus*, as most of these pathogenic isolates were found in the samples (tilapia, rui) of the site (1), which was quite a low-saline site.

In multiple previous studies, *trh*-positive *V. parahaemolyticus* were more frequent than *tdh* positive in the environmental samples (Bauer et al., 2006; Ellingsen et al., 2008). Although only 1–2% of environmental *V. parahaemolyticus* strains harbor *tdh* and/or *trh* gene, this small amount is sufficient to have a significant impact on public health in tropical developing countries (Cook et al., 2002). In our study, 5.26% (17/323) of the *V. parahaemolyticus* strains showed pathogenic potential, by the presence of *trh* gene, but all the isolates were negative for the *tdh* gene. This result appears similar to the previous study by Letchumanan et al. (2015) but contrary to the earlier reports where a high prevalence of these virulence factors was observed in *V. parahaemolyticus* strains in natural estuarine and coastal waters (Johnson et al., 2012; Akther et al., 2016).

In this study, *V. parahaemolyticus* isolates exhibited a high level of resistance to amoxicillin and ampicillin. Resistance to penicillin could probably be due to the extensive use of antibiotics in aquaculture and the effect of residual antibiotics in aquaculture systems. Therefore, the penicillin group is considered ineffective for the management of *V. parahaemolyticus* infections. However, most of the isolates were susceptible to the majority of the antibiotics used. Susceptibility patterns of *V. parahaemolyticus* isolates to different antibiotic groups such as quinolone, tetracycline, carbapenem, aminoglycosides, and cephalosporin were analogous with other studies reported in various sample sources (Lesmana et al., 2001; Han et al., 2007; Yano et al., 2011; Ottaviani et al., 2013). In the current study, the MAR index value was observed greater than 0.2 in 8% (2/17) of pathogenic isolates, which indicates that samples originated from a high-risk contamination source where numerous antibiotics are used (Krumperman, 1983). Aquatic bacteria are subjected to environmental contaminations in coastal and estuarine waters, particularly through wastewater treatment plants and agricultural runoff, which might carry a different amount of antimicrobials and heavy metals and subsequently give selective pressure to the aquatic bacteria to be antimicrobial-resistant (Stepanauskas et al., 2006; Gordon et al., 2007). Therefore, continuous surveillance and monitoring programs are essential for both the prevalence and the antimicrobial susceptibility profile to ensure aquaculture safety.

The pathogenic strains were serotyped for epidemiological purposes. The majority of those strains contained O8 antigen, followed by O5, O11, O3, and O1. Most of the strains could not be typed serologically for the K antigen using conventional kits. In our study, serotype O8: KUT was the most frequent, which was also found in the environmental samples in several other studies (Pal and Das, 2010; Jones et al., 2012; Zhang et al., 2013; Haley et al., 2014). Other serotypes found in this study are O5: KUT, O11: KUT, O3: KUT, O1: KUT, and O3: K20. Interestingly, serotype O1:KUT, a serovariant of O3:K6 (González-Escalona et al., 2008), played a major role in causing a massive outbreak of cholerae in Kolkata, India, and afterward found in Asia, Africa, Europe, Latin America, and the United States (Okuda et al., 1997).

The isolates of this study were categorized into 7 clusters at a similarity of 68% based on the ERIC-PCR approach. The clustering of 5 pathogenic strains out of 17 suggested their genetic linkage though they were collected from different sources at a different time interval. Unlike the other strains in this experiment, the ATCC BAA-238 reference strain only belonged to cluster G. One sediment-derived isolate (R11-40) was placed into the cluster F alone and was distinct from the rest of the isolates genetically. ERIC-PCR-based clustering did not correlate with sources of isolation, time, and serotyping. This finding is similar to that of other research, demonstrating the genetic variation within the strains of *V. parahaemolyticus* (Wong et al., 1999, 2000; Chen et al., 2012; Lu et al., 2016).

Approximately 24% of the pathogenic isolates found in the animal study were associated with fluid accumulation, with/without leading to the death of the rabbit, which implies that all the pathogenic isolates were not able to accumulate fluid in the rabbit intestine. A correlation between enterotoxigenicity of *V. parahaemolyticus* strains and virulence potential was reported in earlier studies (Hiyoshi et al., 2010). Thus, the presence of the *trh* virulence gene could not be linked to enterotoxigenic potential in the present experiment. Xu et al. (1994) showed that the deletion of *trh* led to a minor but noticeable accumulation of fluid in the ligated rabbit small intestine. These findings reveal that *tdh* and *trh* alone do not explain the cytotoxicity and enterotoxicity of pathogenic *V. parahaemolyticus* and propose that certain other mechanisms that have not yet been discovered may contribute to the accumulation of fluid, excluding the factors examined in this study and other studies to date (Park et al., 2004).

This study was limited to only three aquaculture sites due to funding constraints. If an extensive study including a large number of farms were taken, it would be possible to know the scenario better. However, our study provides comprehensive research on pathogenic *V. parahaemolyticus* isolated from different aquacultures by highlighting the occurrence, virulence, antibiotic resistance pattern, serotyping, genetic diversity, and rabbit ileal loop study. This study suggests the presence of pathogenic *V. parahaemolyticus* in the aquaculture sample. Moreover, their resistance to multiple antibiotics could be a threat to public health which should not be ignored. The presence of different serotypes including few pandemic serotypes might indicate the diversity and outbreak potential of these isolates. The clustering of the isolates shows their genetic diversity. The fluid accumulation in the ileal loop of rabbit suggests the pathogenicity of those isolates. These data might be of help in assessing human health risk due to the consumption of fish in the southwestern coastal area of Bangladesh. Subsequently, it can be helpful for public health organizations as a future reference for policymaking guidelines.

## Data Availability Statement

The original contributions presented in the study are included in the article/Supplementary Material, further inquiries can be directed to the corresponding author/s.

## Ethics Statement

The animal study was reviewed and approved by the Animal Experimental Ethics Committee, IRB Secretariat, Research Administration, CMS, International Centre for Diarrhoeal Research, Bangladesh.

## Author Contributions

ZM, MM, SI, and SA: conceptualization. AS, MM, and SI: methodology. MD and MA: software. ZM, RI, DM, and AP: validation and investigation. AS, MM, SA, and MD: formal analysis and data curation. ZM and SI: resources. SA: writing—original draft preparation. MM, AS, ZM, SI, RI, and DM: writing—review and editing. MM, SA, MD, and MA: visualization. ZM: supervision, project administration, and funding acquisition. All authors have read and agreed to the published version of the manuscript.

## Conflict of Interest

The authors declare that the research was conducted in the absence of any commercial or financial relationships that could be construed as a potential conflict of interest.

## Supplementary Material

Supplementary Figure 1 Gel image of species-specific *tlh* positive pathogenic *V. parahaemolyticus.* (Here, serial no represents strain ID in following order: 1 = R01-15, 2 = R01-54, 3 = R01-55, 4 = R06-70, 5 = R06-75, 6 = R06-88, 7 = R06-99, 8 = R06-100, 9 = R06-101, 10 = R06-102, 11 = R06-106, 12 = R06-107, 13 = R09-14, 14 = R09-20, 15 = R10-16, 16 = R11-40, and 17 = R12-76.
