# Supplementary figures and images for: Characterization of Pathogenic Vibrio parahaemolyticus Isolated From Fish Aquaculture of the Southwest Coastal Area of Bangladesh
Source: Front Microbiol. 2021 Mar 8;12:635539. doi: 10.3389/fmicb.2021.635539 (PMC7982743; doi:10.3389/fmicb.2021.635539)

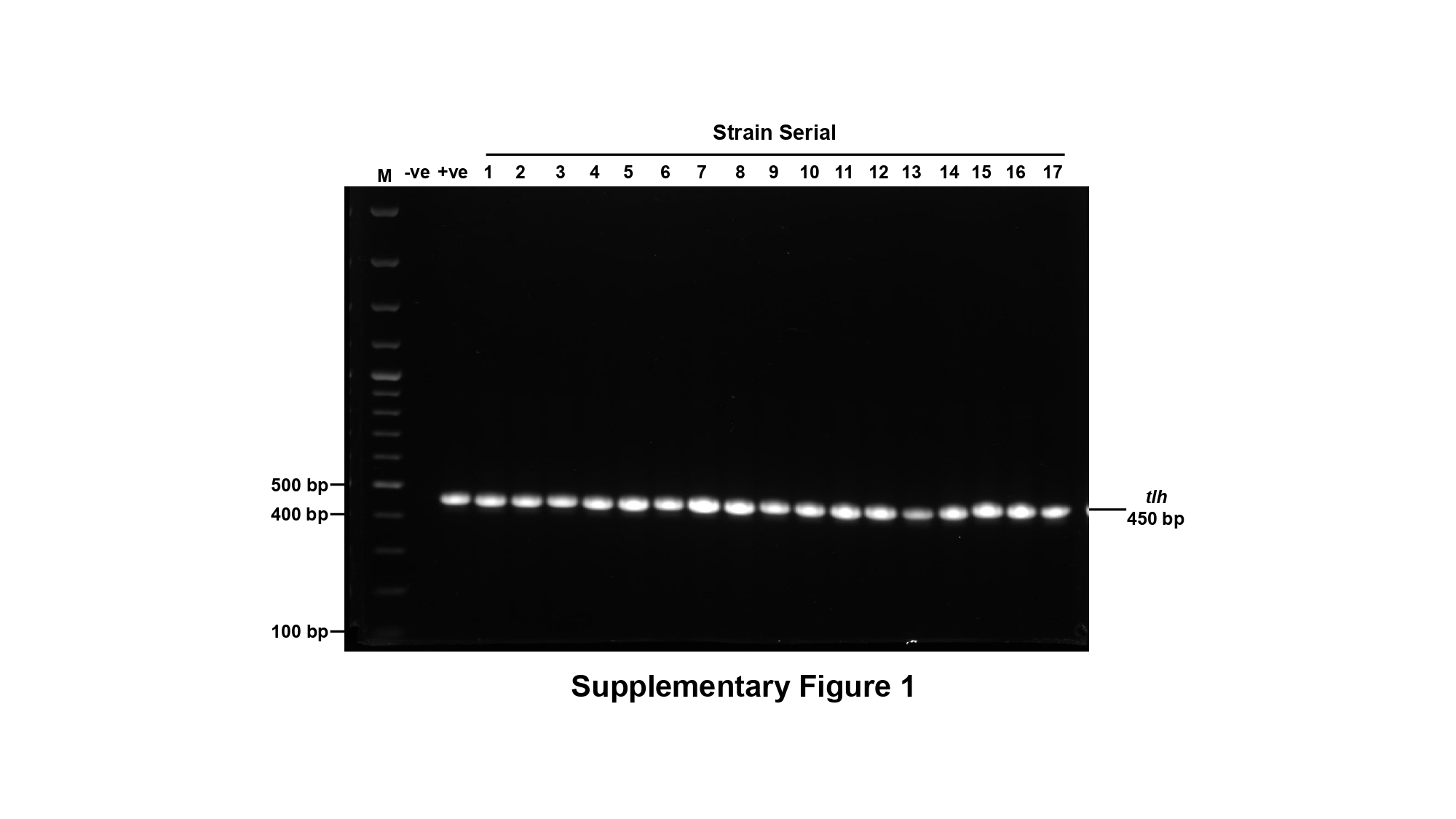

Supplement: Supplementary Figure 1 — Gel image of species-specific tlh positive pathogenic V. parahaemolyticus. (Here, serial no represents strain ID in following order: 1 = R01-15, 2 = R01-54, 3 = R01-55, 4 = R06-70, 5 = R06-75, 6 = R06-88, 7 = R06-99, 8 = R06-100, 9 = R06-101, 10 = R06-102, 11 = R06-106, 12 = R06-107, 13 = R09-14, 14 = R09-20, 15 = R10-16, 16 = R11-40, and 17 = R12-76. [file Image_1.JPEG]
